# Cardiac sarcomere mechanics in health and disease

**DOI:** 10.1007/s12551-021-00840-7

**Published:** 2021-10-12

**Authors:** Claudia Crocini, Michael Gotthardt

**Affiliations:** 1 Max Delbrück Center for Molecular Medicine in the Helmholtz Association (MDC), Neuromuscular and Cardiovascular Cell Biology, Berlin, Germany; 2 German Center for Cardiovascular Research (DZHK) Partner Site Berlin, Berlin, Germany; 3 BioFrontiers Institute & Department of Molecular and Cellular Development, University of Colorado Boulder, Boulder, USA; 4 Charité-Universitätsmedizin Berlin, 10117 Berlin, Germany

**Keywords:** Cardiac sarcomere mechanics, Cardiac disease, Titin

## Abstract

The sarcomere is the fundamental structural and functional unit of striated muscle and is directly responsible for most of its mechanical properties. The sarcomere generates active or contractile forces and determines the passive or elastic properties of striated muscle. In the heart, mutations in sarcomeric proteins are responsible for the majority of genetically inherited cardiomyopathies. Here, we review the major determinants of cardiac sarcomere mechanics including the key structural components that contribute to active and passive tension. We dissect the molecular and structural basis of active force generation, including sarcomere composition, structure, activation, and relaxation. We then explore the giant sarcomere-resident protein titin, the major contributor to cardiac passive tension. We discuss sarcomere dynamics exemplified by the regulation of titin-based stiffness and the titin life cycle. Finally, we provide an overview of therapeutic strategies that target the sarcomere to improve cardiac contraction and filling.

## Introduction

**Fig. 1:**
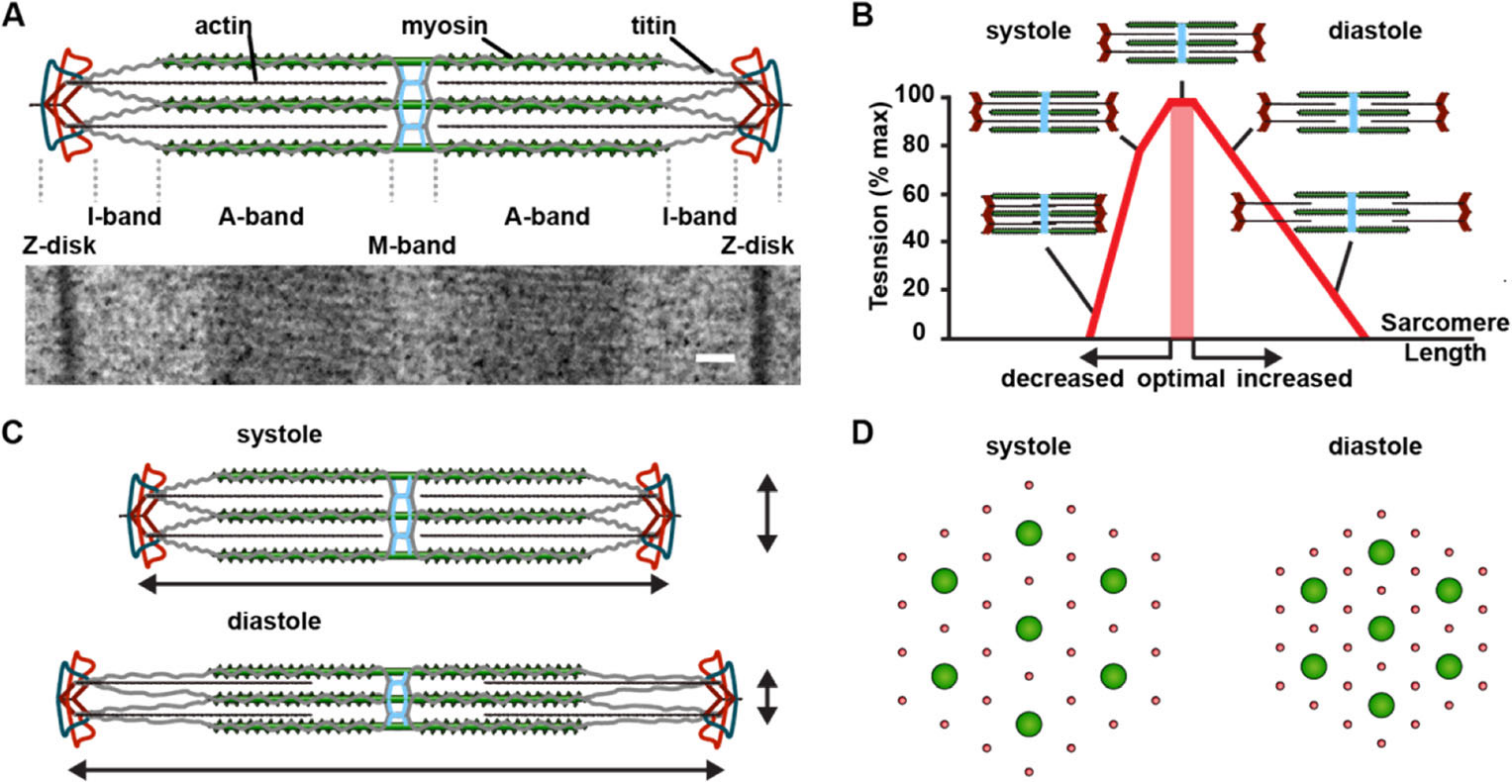
Sarcomeric structure-function relations. **A** Schematic of sarcomere (above) and sarcomere ultrastructure observed by electron microscopy (below). The sarcomere is an elastic scaffold that consists of structural proteins lined out from Z-disk to M-band, including actin (black), myosin (green), and titin (grey) that extends through the half-sarcomere. Scale is 100 nm. **B**Length-tension relation, a contributor to the Frank-Starling Law of the heart. Adapted from (Gordon et al. 1966). **C** Sarcomere length changes during systole and diastole. **D** Myofilament compressions during diastolic filling, Myosin in green and actin in red

Cardiac diseases are the leading cause of death for both men and women in Western countries (Who 2019) and are often characterized by alterations of *active forces*(contraction) and/or*passive forces* (myocardium passive tension) (van der Velden and Stienen 2019). The main source of both active and passive tension is the *cardiomyocyte*. Cardiomyocytes represent about 50% of the cells in the human heart ventricles (Litvinukova et al. 2020). They are specialized striated muscle cells that actively generate force, pumping blood into the vascular system, and then relax allowing the passive filling of the ventricles up to the limits provided by the passive tension of the organ. Within the cardiomyocyte, *sarcomeres* are responsible for the generation of active and passive forces. Sarcomeres are highly ordered multiprotein complexes longitudinally aligned to give the characteristic striated look of cardiomyocytes and skeletal muscle cells (Figure 1A). Active forces are generated by actins and myosins sliding along each other in a process that involves *cross-bridge* formation, while passive tension in the sarcomere is generated by the giant spring protein titin (Maruyama et al. 1977a; Maruyama et al. 1977b; Horowits and Podolsky 1988). Titin is the largest protein in the human proteome, and its homeostasis represents a major challenge for muscle cells. In addition to actin, myosin, and titin, which make up the thin, thick, and elastic filament system, respectively, more than 200 proteins populate the cardiac sarcomere and are continuously and dynamically assembled, modified, and degraded to maintain and adapt cardiac function (Martin and Kirk 2020). Structural and functional alterations of the sarcomere and its regulators underlie a wide spectrum of different cardiac diseases, and it is therefore of critical importance to understanding cardiac sarcomere mechanics. In this review, we revisit the main determinants of sarcomere mechanics, distinguishing between active and passive forces. We discuss the structure of thin, thick, and titin filaments of the sarcomeres; the cross-bridge formation; and the importance of other sarcomere-associated proteins. We then describe the Frank-Starling mechanism and Ca^2+^ homeostasis as major determinants for sarcomere activation and the interrelation of diastolic filling and systolic ejection. We explore titin as it is the source of passive tension within the sarcomere and discuss current knowledge about titin life cycle as a paradigm for sarcomere homeostasis. Finally, we discuss current and potential therapeutic strategies targeting the sarcomere with examples for both active and passive forces. Overall, we aim to provide insights into the complexity of sarcomere mechanics and its relevance to cardiac disorders.

### Sarcomere structure and protein composition

At its core, the mature cardiac sarcomere is a regular hexagonal lattice of thin, actin-containing filaments attached to the Z-disk and thick, myosin-containing filaments interconnected in the middle of the sarcomere via the M-band (Figure 1A). In addition, the filamentous protein titin spans the half-sarcomere from the Z-disk to M-band and stabilizes contraction, among other functions discussed below. The structural properties of sarcomeric proteins and their arrangement are central to cardiac contraction. Sarcomeres are connected in series at the Z-disk and arranged in parallel to form bundles of myofibrils about 1 μm in diameter. Sliding of thick and thin filaments relative to each other results in muscle contraction.

### Thick and thin filaments

The thick filament mainly consists of myosin molecules. The myosin superfamily encodes 18 classes of myosin motors, which are ubiquitous in eukaryotes and participate in several cellular motile processes (Hartman and Spudich 2012). A subset of class II myosins power muscle contraction in striated muscles, MYH6 (α-MyHC) and MYH7 (β-MyHC), are known as the cardiac myosin heavy chain isoforms and, albeit 93% identical in humans, display significantly different functional properties. α-MyHC has a higher ATPase activity but generates less force than β-MyHC (Pope et al. 1980; Aksel et al. 2015). In the adult human ventricle, the cardiac myosin composition is 95% β-MyHC and 5% α-MyHC (Reiser et al. 2001), a ratio that further changes in favor of β-MyHC in cardiac diseases (Bouvagnet et al. 1989; Nadal-Ginard and Mahdavi 1989). There are two functional units in class II myosins, a globular motor domain (myosin head) that contains the catalytic ATPase site and binds actin and an α-helical coiled-coil rod domain that dimerizes and assembles into bipolar thick filaments. In the center of the thick filament, the bare zone is free of myosin heads as a consequence of the bipolar arrangement of the myosin molecules. Three-dimensional studies on tarantula thick filaments showed that two myosin heads pack together to form an interacting-heads motif (IHM) (Woodhead et al. 2005). The IHM forms only in relaxed muscle and is an evolutionarily conserved motif among species and muscle types (Alamo et al. 2016). Relaxed myosin exists in two conformations: disordered relaxation (DRX), with one of the two paired myosin heads folded (blocked head) and super relaxation (SRX), with both heads folded back along the thick-filament backbone. As compared to the DRX conformation, myosins in SRX do not participate in contraction and conserve energy, while providing reserve heads that can be activated in response to increased mechanical need (McNamara et al. 2015). The thick filaments contain cardiac myosin-binding protein-C (cMyBP-C) (Carrier et al. 2015), which resides in the A band and interacts with myosin (Starr and Offer 1978; Alyonycheva et al. 1997) and titin (Labeit et al. 1992; Soteriou et al. 1993) and helps maintain sarcomeric structure and regulates cardiac contraction and relaxation.

The thin filament consists of a double-stranded helix of G-actin also called filamentous actin (F-actin), two tropomyosin (Tm) strands, and the three troponin (Tn) subunits, TnT, TnI, and TnC, which together form the Ca^2+^-regulatory complex of the thin filament. Tm strands lie within the two grooves of F-actin and provide the thin filament with stability, flexibility, and cooperativity. The adult human heart expresses about 80% α-cardiac actin and 20% α-skeletal actin encoded by *ACTC* and *ACTA1* genes, respectively. Higher expression of α-skeletal actin is associated with increased contractility in mouse (Hewett et al. 1994) and in diseased human hearts (Copeland et al. 2010). Myosin thick filaments and actin thin filaments interdigitate and slide past one another to cause sarcomere shortening (Powers et al. 2021). At the functional level, the myosin head directly interacts with two adjacent actin monomers (Behrmann et al. 2012). This attachment of myosin to actin is a multi-step process (Holmes et al. 2004; Behrmann et al. 2012) that starts with calcium binding to TnC, resulting in reduced affinity of TnI for actin (da Silva and Reinach 1991) and exposing myosin-binding sites on actin (McKillop and Geeves 1993). Myosin binding to actin further displaces tropomyosin and prevents its return into the blocking position (Xu et al. 1999). When calcium dissociates from troponin, myosin heads detach from actin progressively, allowing Tm and the troponin complex to move back to an inhibitory position on the thin filament.

### Titin filaments

Within the sarcomere, the giant protein titin spans from the Z-disk to the M-band and functions as a scaffold and molecular spring. Titin is encoded by a single gene of 363 exons, and its passive tension can be adjusted in the long term by titin isoform switching via alternative splicing — e.g., peri- and postnatally to meet the increased needs of the developing heart — or more readily by post-translational modifications. The titin region located in the I-band is extensible and consists of immunoglobulin-like (Ig) domains arranged in tandem, the PEVK sequence (rich in proline, glutamate, valine, and lysine residues), and the N2B element. Each functions as a distinct spring element in series. The portion of titin located in the A band is inextensible and composed of regular Ig and fibronectin type 3 (Fn3) domains that form so-called super-repeats. The C-terminal region of titin is located at the M-band and contains a kinase domain, while the N-terminus is in the Z disk of the sarcomere. Titin filaments with opposite polarity overlap and interconnect at both Z-disk and M-band, forming a contiguous filament along the myofibril. Single-molecule studies using laser tweezers and atomic force microscopy (Kellermayer et al. 1997; Li et al. 2002; Watanabe et al. 2002a; Watanabe et al. 2002b) have shown that titin can be compared to a modular polymer of connected elastic segments, each with distinct extensibility, acting as a multistage spring in response to stress applied in the axial direction. Without external force, titin behaves as an entropic spring, where each “module” of the spring is folded and resists stretch. When external force is applied, titin domains unfold and participate in the entropic elasticity of the newly adjusted spring characterized by an increased length and a different spring constant. Upon relaxation, each module folds individually. The unfolding/refolding order is determined by the intrinsic properties of folding pathway for each individual titin domain. Immunolabeling of selected titin domains in rodent left ventricular myocardium revealed that tandem Ig domains in the I-band are extended first, followed by the PEVK segment, and lastly the N2B segment (Linke et al. 1999; Trombitas et al. 1999; Trombitas et al. 2000). As a result, sarcomere passive tension rises slowly at first and then exponentially — limited by the extension of the I-band domains, the rupture of interfilament interactions, and the resistance of non-extensible regions. In fact, Ig and Fn3 domains in the A-band region of titin do not unfold/refold significantly during physiological contraction cycles, because of the strength of interaction between titin and the thick filaments (Wang et al. 1991, 1993). This feature preserves the efficiency of the sarcomere and may function as a molecular ruler, regulating assembly of the thick filament.

### Accessory proteins of the sarcomere

A large number of accessory proteins are present within the sarcomere and support the structural, mechanical, signaling, and transport functions. These proteins populate predominantly the Z-disk and the M-band of the sarcomere and are often characterized by a dynamic sarcomeric and/or cellular localization in response to intra- and extra-cellular signals (Lange et al. 2006). For instance, ubiquitin E3 ligases MURF1/2/3, FHL protein family, and CARP are located in the cardiac sarcomere and in the nucleus. MURFs are present at both the M-band and Z-disk suggesting a specialized location-dependent role and can translocate to the nucleus and participate to cardiac transcriptional regulation (Willis et al. 2009; Perera et al. 2011; Willis et al. 2014). FHL2 is highly expressed in the heart (Scholl et al. 2000) and present in two regions of the sarcomere (I-band and M-band), as well as the nucleus, and focal contacts. CARP localizes at the I-band and Z-disk and acts predominantly as a corepressor of transcription through interaction with the ubiquitous transcription factor YB-1 (Jeyaseelan et al. 1997; Zou et al. 1997). Recently, the complexity of the protein networks that connect to the sarcomere Z-disk has been explored using biotin ligase (BioID) inserted at titin’s Z-disk region, providing a census of the sarcomeric proteome in the heart and skeletal muscle in vivo (Rudolph et al. 2020). This approach was used in neonatal and adult heathy mice but could be extended to other sarcomeric regions and pathophysiological states.

### Sarcomeric cardiomyopathies

Several genetic cardiac diseases result from mutations in genes that encode for sarcomere proteins. Particularly, hypertrophic cardiomyopathy (HCM) and dilated cardiomyopathy (DCM) are the two predominant types of cardiomyopathy and are highly variable both genetically and phenotypically (Masarone et al. 2018). HCM is characterized by preserved or elevated systolic function but diminished relaxation resulting from asymmetric thickening of the ventricular walls, cardiac fibrosis, and cardiomyocyte disarray (Maron 2010; Harvey and Leinwand 2011). Conversely, in DCM patients, systolic performance is reduced, and the ventricle is dilated rather than hypertrophic (Hershberger et al. 2013; McNally et al. 2013). Both HCM and DCM have been linked to hundreds of sarcomeric gene mutations and different pathogenic mechanisms (Yotti et al. 2019). Generally, sarcomeric proteins with missense mutations are expected to incorporate into the sarcomere and contribute to disease by disrupting normal mechanical function. Conversely, gene mutations as insertions, deletions, premature stop codons, or altered splice sites likely result in unstable proteins that degrade prematurely and cause cardiomyopathies through a haploinsufficiency mechanism (Marston et al. 2009; Kampourakis et al. 2018). HCM mutations reside mainly in MYH7 and MYBPC3, and more rarely in MYL2 or MYL3, which encode the ventricular myosin regulatory light chain (RLC) or myosin essential light chain (ELC), respectively (Richards et al. 2015; Marian and Braunwald 2017). Most HCM mutations in MYH7, MYL2, and MYL3 alter residues that participate in IHM and change the charge of the encoded amino acid, possibly resulting in destabilization of SRX myosin heads and an increased proportion of myosin heads in DRX (Alamo et al. 2017). MYBPC3 mutations associated with HCM have also been linked to changes in the myosin DRX/SRX-ratio in human hearts (McNamara et al. 2017; Toepfer et al. 2019). This is likely the result of intermyofilament interactions between cMyBP-C and myosin that have been recently assessed with unprecedented resolution in the heart (Brunello et al. 2020). A shift in balance favoring DRX over SRX would increase the number of available heads for actin interactions, resulting in hypercontractility, impairment of relaxation, and increased energy consumption, all hallmarks of HCM. A different mechanism is likely responsible for HCM missense mutations occurring in the rod domain of β-MyHC (Blair et al. 2002; Waldmuller et al. 2002; Richard et al. 2003; Karkkainen et al. 2004; Van Driest et al. 2004; Hougs et al. 2005; Perrot et al. 2005). These mutations are located too far from the IHM and the head to affect myosin SRX-DRX ratio or myosin motor and ATPase function but can impair assembly and stability of myofilaments (Buvoli et al. 2008; Armel and Leinwand 2009, 2010; Wolny et al. 2013). Thin filament mutations associated with HCM are predominantly located in TPM1 (α-tropomyosin), TNNT2 (TnT), TNNI3 (TnI), and ACTC1, resulting in a phenotypically distinct class of patients with increased risks for cardiac dysfunction and heart failure (Coppini et al. 2014). These mutations lead to increase calcium sensitivity and altered response to signaling pathways, which enhances contractility but impairs relaxation (Cheng and Regnier 2016; Gangadharan et al. 2017).

As DCM is characterized by the reduced mechanical force generation, pathogenic gene mutations (Kamisago et al. 2000; McNally et al. 2013) result generally in opposite molecular mechanisms as compared to HCM. In fact, DCM mutations in MYH7 reduce myosin ATPase activity and motor function (Schmitt et al. 2006), while DCM mutations in thin-filament proteins decrease myofibril calcium sensitivity, resulting in reduced tension and faster relaxation (Robinson et al. 2007; Gangadharan et al. 2017). Approximately 90% of titin mutations are associated with DCM phenotype, and the remaining to HCM (Greaser 2009). Specifically, mutations that lead to titin truncated variants are highly associated with DCM (Herman et al. 2012; Merlo et al. 2013; Tharp et al. 2019). Integrated analysis of sequencing and transcriptional data from large human cohorts has demonstrated that the effect of titin-truncated variants is dependent on the position of the truncation within the protein (Roberts et al. 2015). The majority of DCM patients carry titin-truncated variants located in the A-band (Herman et al. 2012; Roberts et al. 2015; Akinrinade et al. 2016; Schafer et al. 2017), but in general, truncations occurring in constitutive (highly expressed) exons of titin lead to DCM (Roberts et al. 2015). Mechanisms by which titin-truncating variants cause DCM are probably related to haploinsufficiency rather than dominant negative effects. In fact, truncated titin peptides are not found in DCM hearts (Roberts et al. 2015) likely due to nonsense-mediated decay and rapid turnover of the mutant peptides (Schafer et al. 2017). Accessory proteins of the sarcomere contribute to cardiac contraction and are linked mechanically to thin, thick, and titin filaments as well as additional non-sarcomeric compartments. Mutations of these proteins can also lead to HCM and DCM (Selcen and Carpen 2008; Lange et al. 2020; Wadmore et al. 2021) and have shown to exhibit altered contractility via different mechanisms (Adams et al. 2007; Friedrich et al. 2012; Crocini et al. 2013).

## Active forces

The contractile function of the heart is intimately related to the mechanical properties of sarcomeres as their building blocks and is determined by structural parameters of orientation and density of cardiac sarcomeres and by temporal parameters affecting sarcomere activation and relaxation.

### The Frank-Starling mechanism

Cardiac contraction and shortening dynamically adjusts on a beat-to-beat basis in response to changes during cardiac filling. This phenomenon is historically referred to as the Frank–Starling Law of the heart (Zimmer 2002; Shiels and White 2008; Sequeira and van der Velden 2015). The law describes the direct relationship between length of the cardiac fibers and the force generated in contraction. This mechanism links changes in cardiac filling to the subsequent ejection of blood into the circulation: an increase in venous return dilates the ventricles, stretching the myocardium that respond by increasing both contractility and stroke volume. In this way, the Frank–Starling mechanism is central to the regulation of cardiac output. For decades, the underlying mechanism for the Frank-Starling Law was considered the degree of overlap between the actin and myosin filaments, or sarcomere length, which would guarantee maximum number of cross-bridges (Gordon et al. 1966). The development of active tension is optimal near the resting sarcomere length, ~ 2 μm, and decreases at higher or lower sarcomere lengths. One can define a descending limb of the isometric length–tension relation, at sarcomere lengths above 2.2 μm, and an ascending limb of the isometric length–tension relation, at sarcomere lengths below ~ 2 μm (Figure 1B). The cardiac sarcomere operates between ~ 2 and 1.8 μm (Spotnitz et al. 1966), i.e., in the ascending limb of the isometric length–tension relation, in which active tension increases with an increase in sarcomere length. Increased diastolic filling results in an increase in sarcomere length and thus an increase in the tension-generating capacity as more myosin heads within the cardiac myocytes are able to bind to actin (Figure 1C). However, a significant increase in active tension occurs over the region 2.0–2.2 μm in mammals (Fabiato and Fabiato 1975; Allen and Kentish 1985), suggesting that myofilament overlap cannot completely account for the length–tension relation in cardiac muscle (Jewell and Wilkie 1960; Hill 1964). Titin has been suggested to play a major role in the length-dependent activation of active force in the heart. Radial forces generated by titin would reduce lateral spacing between thick and thin filaments at higher sarcomere length (Figure 1C, D) that may also influence the affinity of Ca^2+^ ions to the negatively charged myofilaments (McDonald and Moss 1995; Wang and Fuchs 1995). This would at least in part explain direct relationship between myofilament length and their sensitivity to Ca^2+^ ions, as indicated in the Frank-Starling Law. Several other structural and molecular mechanisms are likely participating to the Frank-Starling Law and include post-translation modifications of myofilament proteins and sarcomere-independent components (Sequeira and van der Velden 2017). Although a unifying idea that describes the Frank–Starling Law of the heart is still missing, it is an essential component for the cardiac function, and its understanding could help develop therapeutic strategies for human heart disease.

### Calcium homeostasis

Cytosolic calcium concentration is fundamental for mechanical activation of the sarcomere. At rest, intracellular calcium concentration is at submicromolar level but rises during the plateau phase of the cardiac action potential, thanks to the cardiac-specific voltage-sensitive L-type Ca^2+^ channels. This unique cardiac current triggers release of additional calcium from the sarcoplasmic reticulum via the ryanodine receptor (RyR, calcium-induced calcium release) (Bers 2002) that activates contraction. Collectively, this process is called excitation–contraction (E-C) coupling (Fozzard et al. 1992; Bers 2002; Page, 2002). The level of sarcomere activation is not simply proportional to cytosolic calcium concentration, but rather it is the result of a complex and dynamic signaling process that is regulated by numerous factors (de Tombe 2003). However, there is generally a direct relationship between the magnitude of the calcium transient and the sarcomere (ventricle) contraction (Gwathmey and Hajjar 1990; Backx et al. 1995; Bassani et al. 1995), thus making calcium one of the major determinants of heart contractility. Altered intracellular Ca^2+^ handling underlies numerous cardiac diseases. In heart failure, for example, E-C coupling is affected by functionally defective L-type Ca^2+^ channels (Piot et al. 1996; Barrere-Lemaire et al. 2000; He et al. 2001) and increased space between L-type Ca^2+^ channels and the RyR (Gomez et al. 1997), as well as decreased Ca^2+^ content in the sarcoplasmic reticulum (Lindner et al. 1998; O'Rourke et al. 1999; Hobai and O'Rourke 2001) and altered channel-gating property of RyR (Yamamoto et al. 1999; Ono et al. 2000; Yano et al. 2000; Marx et al. 2001). Sarcomere activation not only depends on the amount of Ca^2+^ released at each given heartbeat but also on the rate of Ca^2+^ release across the cell (Yano et al. 2005). To ensure a synchronized activation of all sarcomeres within each cell, mammalian cardiac myocytes are provided with a complex network of membrane invaginations called the *transverse-axial tubular system* or *t-tubules*(Ferrantini et al. 2013). T-tubules allow for rapid and homogenous propagation of the cardiac action potential, thus Ca^2+^ entry, and affect cardiac sarcomere mechanics. Loss of t-tubules by acute formamide-induced osmotic shock (detubulation) induces prolonged contraction kinetics and impaired force–frequency response and can be, at least in part, mitigated by improving Ca^2+^ synchrony and propagation (Ferrantini et al. 2014). T-tubular structural remodeling reduces Ca^2+^ synchrony in failing human hearts (Louch et al. 2004) and murine cardiac disease models (Louch et al. 2006; Wei et al. 2010; Heinzel et al. 2011), and also, t-tubule functional remodeling (Crocini et al. 2017) can affect Ca^2+^ propagation and contraction (Crocini et al. 2014; Crocini et al. 2016a; Crocini et al. 2016b; Scardigli et al. 2018).

## Passive forces

When cardiac muscle is stretched beyond its resting sarcomere length, it develops passive tension. Passive tension is essential for the heart as it contributes to the diastolic wall tension that determines the extent of filling of the ventricle and the subsequent stroke volume (for reviews, see Allen and Kentish 1985; Brady,1991a). Passive tension is also important in the activated myocardium because it participates in determining the shortening velocity of cardiac trabeculae (de Tombe and ter Keurs 1992) and cardiac myocytes (Sweitzer and Moss 1993). Over the working range of the heart (sarcomere lengths 1.8-2.2 μm), collagen and titin are the most important contributors to passive tension, with collagen dominating at the longer sarcomere lengths of the working range and titin at the shorter lengths (Linke et al. 1994; Granzier and Irving 1995). Collagen is a fibrous protein of the extracellular matrix packed to form long, thin, and strong fibrils. Notably, cardiac fibrosis with altered collagen expression and composition is a hallmark of many cardiac diseases and can dramatically affect cardiac contraction and relaxation (Travers et al. 2016). Minor contributions are also provided by intermediate filaments, measured as ~ 10-fold less than titin, and even less by microtubules. Here, we focus on the titin protein as it represents the sarcomeric source of passive tension.

### Titin-based passive tension

**Fig. 2:**
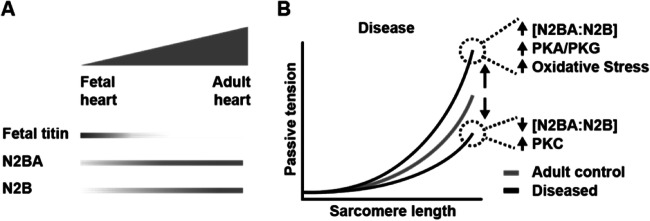
Titin-based passive tension. **A** Developmental changes in titin isoform expression facilitate the transition from fetal to adult force generation. **B** The relationship of sarcomere length and passive tension changes in disease via posttranslational modifications (protein kinases PKA, PKC, PKG acting on the titin spring region) and changes in titin isoform expression (N2BA to N2B titin isoform ratio)

As mentioned, titin-based stiffness can be adjusted both in the long term by titin isoform switching (Granzier and Irving 1995; Opitz et al. 2004) or more rapidly by post-translational modifications (Koser et al. 2019). Abnormal isoform ratios or post-translational modifications can dramatically affect passive tension of cardiomyocytes and have been described in many human cardiac diseases (LeWinter and Granzier 2014). In the heart, there are 3 main types of titin isoforms: fetal cardiac titin, adult N2BA, and adult N2B. They differ in their I-band extensible regions. Fetal cardiac titin isoforms are longer and more compliant than either N2B or N2BA (Lahmers et al. 2004; Opitz et al. 2004) (Figure 2A). These isoforms could be beneficial in fetal-neonatal development because of the low filling pressure of the fetal heart and the structural constraints provided by other tissues that limit cardiac reserve in the fetus (Walker and de Tombe 2004). Fetal isoforms gradually disappear during postnatal development in favor of the mature N2BA and N2B. Titin N2BA isoforms have a longer PEVK sequence and a variable number of additional Ig domains resulting in more compliant isoforms than N2B titin. Both isoforms are co-expressed in the cardiac sarcomere, and their ratio is a determinant of passive stiffness. In adult human left ventricle, the N2BA/N2B ratio is ~0.6 and can change in disease (Neagoe et al. 2002; Makarenko et al. 2004; Nagueh et al. 2004) (Figure 2B). The N2BA:N2B ratio is increased in DCM patients (Nagueh et al. 2004) and would result in reduced passive tension leading to reduced diastolic forces and dilation of the heart, both hallmarks of DCM. Switching to the longer N2BA isoform could represent an initial compensatory mechanism that improves diastolic function; however, long-term reduction of passive tension and diastolic pressure could worsen contractile performance in systole (Makarenko et al. 2004).

The RNA-binding motif 20 (RBM20) is a well-characterized regulator of cardiac isoform expression for titin and over 30 additional genes (Guo et al. 2012; Maatz et al. 2014; van den Hoogenhof et al. 2018; Lennermann et al. 2020). RBM20 is regarded as a splicing repressor. In the case of titin pre-mRNA, RBM20 binds and represses splicing of large stretches of exons, allowing alternative splice sites at the 3′ or 5′ end of RBM20-repressed regions to splice together (Li et al. 2013). However, loss of RBM20 function leads to larger titin proteins, with reduced N2B isoform and upregulation of N2BA isoform and another, giant, and even more compliant titin isoform called N2BA-G. As a result, myocardial stiffness is reduced, and resting sarcomere length is increased (Greaser et al. 2008; Guo et al. 2012; Methawasin et al. 2014; Beqqali et al. 2016; Methawasin et al. 2016). RBM20 mutations lead to a severe form of DCM with high rates of heart failure, arrhythmias, and sudden cardiac death (Brauch et al. 2009; Li et al. 2010; Wells et al. 2013; van den Hoogenhof et al. 2018), a complex phenotype resulting from missplicing of several RBM20 target genes (Guo et al. 2012; Maatz et al. 2014). In particular, missplicing of the RBM20-target calcium/calmodulin-dependent protein kinase II (CaMKII), a crucial modulator of E-C coupling in cardiomyocytes (Maier and Bers 2007), is likely responsible for altered cellular calcium handling and cellular calcium overload observed in RBM20 cardiomyopathy (van den Hoogenhof et al. 2018).

In order to meet beat-to-beat requirements of the heart, titin tension can be modulated via post-translational modifications of titin spring elements (Koser et al. 2019), especially N2B and PEVK unique sequences. A few kinases have been implicated in titin phosphorylation, including protein kinase A (PKA), protein kinase G (PKG), and CaMKII (Kruger et al. 2009; Hamdani et al. 2013; Kotter et al. 2013; Koser et al. 2019). PEVK phosphorylation (via CaMKII) increases titin stiffness, while N2B phosphorylation (via PKG and PKA) decreases it. Abnormal regulation of signaling pathways has been found in numerous cardiac diseases and can affect titin passive tension. For example, human failing hearts show increased cardiomyocyte passive tension via hyperphosphorylation of PEVK and reduced phosphorylation of N2B (Hopf et al. 2018). Targeting intracellular pathways holds very promising therapeutic potential and has been already validated in animal models (Eisenberg et al. 2016; Hopf et al. 2018; Leite-Moreira et al. 2018; Slater et al. 2019).

### Titin life cycle

Considering the impressive molecular weights of titin and the plethora of possible isoforms, it could be tempting to think that titin protein homeostasis is a static and slow process. However, titin dynamics studies revealed an unexpected level of protein mobility in and out of sarcomeres (da Silva et al. 2011; Rudolph et al. 2019; Cadar et al. 2020). Pulse-chase experiments demonstrated that the protein half-life of titin is approximately 70 h in cultured skeletal muscle cells (Isaacs et al. 1989). Using isolated neonatal cardiomyocytes from a titin-eGFP knock-in mouse model in combination with fluorescent recovery after photobleaching (FRAP) imaging, a later work (da Silva et al. 2011) reported that the exchange half-life for titin in the sarcomere is 2.1 h. Taken together, these two works would suggest the existence of a pool of sarcomeric and non-sarcomeric titin molecules that can rapidly exchange in the sarcomere as they move within hours and are replaced within days. Titin synthesis, motility, and sarcomeric (dis)integration have been further resolved using FRAP and a bifluorescently labeled knock-in mouse to simultaneously visualize both ends of titin molecules (Rudolph et al. 2019). This work demonstrated that there is a pool (> 15%) of soluble titin molecules readily available for integration into the sarcomere. Additionally, this work showed that titin is inserted into the mature sarcomere stochastically from either ends of the protein — starting at the Z-disk or at the M-band — in contrast with myofibrillogenesis predictions (Rhee et al. 1994; Du et al. 2008). Titin dynamics differed considerably between embryonic and mature cardiomyocytes, highlighting the complexity of cardiac sarcomere assembly and roles at various life stages.

## The sarcomere as a therapeutic target

There is a remarkable growth of knowledge about sarcomere mechanics and how active and passive forces are regulated in disease states. Translation of these findings to the clinics has been facilitated by both technological advances in model systems and analysis tools, including the generation of small and large animal models (e.g., using CRISPR/Cas9 technology) and the advent of induced pluripotent stem cell (iPSC) technology. The differentiation of iPSCs into cardiomyocytes has seen tremendous advances (Hirt et al. 2014; Ronaldson-Bouchard et al. 2018), and efforts combining iPSC-derived cardiomyocytes with clinically relevant animal models are generating fundamental insights to understanding mechanisms of cardiac pathology and to exploring different therapeutic strategies (Coppini et al. 2014). For instance, a functional readout has been developed to study TnT mutations leading to HCM or DCM (Pettinato et al. 2020), RBM20 therapy has been validated in a DCM model (Briganti et al. 2020), and disruption of SRX state has been linked to hypercontractility in cells harboring a MYH7 mutation (Vander Roest et al. 2021). These new experimental tools confirm that the sarcomere is an excellent therapeutic target (Table 1) — due to both the number of mutations in sarcomeric proteins causing human disease and because of its unique role in determining systolic and diastolic properties. Therapeutic approaches that directly target the primary cause of inherited cardiomyopathies, i.e., genetic mutations of sarcomere proteins, are limited, but targeting the sarcomere has shown promising results in ameliorating a wide variety of cardiomyopathies. We discuss here sarcomere targets and potential therapies distinguishing between active and passive forces.

**Table 1:** Sarcomere targets and potential therapies

**Target**	**Potential therapy**
***Active forces***	
Mutant genes	Gene therapy
•cMyBPC	•Trans-splicing (Mearini et al. 2013)
•cMyBPC	•mRNA silencing (Gedicke-Hornung et al. 2013)
•cMyBPC	•Gene replacement (Mearini et al. 2014)
Myofilament Ca^2+^ sensitivity	Ca^2+^ desensitizers
	•Catechins (Adhikari and Wang 2004)
•cTnC	•Calmodulin antagonists (Hidaka et al. 1980; Silver et al. 1986; Osawa et al. 1998)
	•β-blockers: nebivolol (Zeitz et al. 2000)^b^
•cTnI	•β3 adrenergic receptor agonist (Lee et al. 2010)
Myosin	Small molecules
	•Myosin activator: omecamtiv mecarbil (Teerlink et al. 2021)^a^
	•Myosin inhibitor: mavacamtem (Olivotto et al. 2020)^a^
	Genetic approaches
	•Allele specific mRNA silencing (Jiang et al. 2013) •Isoform composition: lnRNA Myh7b (Broadwell et al. 2021)
***Passive forces***	
Titin	Small molecules
•Titin phosphorylation	•Phosphodiesterase-5 inhibitor: sildenafil (Redfield et al. 2013; Hoendermis et al. 2015)
•Titin phosphorylation	•Phosphodiesterase-9 inhibitor (Lee et al. 2015)
•Titin phosphorylation	•β3 adrenergic receptor agonist (Lee et al. 2010)
•Titin phosphorylation	•Metformin (Slater et al. 2019)
	Genetic approaches
RBM20	Gene therapy
	•RBM20 modulation (Methawasin et al. 2014; Hinze et al. 2016; Methawasin et al. 2016; Pulcastro et al. 2016)

^a^In clinical trial

^b^In the clinics

### Targeting active forces

Genetic approaches to revert sarcomere mutations have been explored in animal models and iPSC-derived cardiomyocytes (Gedicke-Hornung et al. 2013; Jiang et al. 2013; Mearini et al. 2013; Prondzynski et al. 2017; van Kampen and van Rooij 2019), but translation to the patient could be challenging due to possible off-target effects of gene-editing, invasiveness, and poor efficiency of delivery methods. To date, cardiac gene therapy in adult heart failure patients has been attempted with an adeno-associated viral vector encoding the non-sarcomeric gene SERCA2a under control of a cytomegalovirus promoter and intracoronary infusion (Jessup et al. 2011; Zsebo et al. 2014; Greenberg et al. 2016; Hulot et al. 2017; Lyon et al. 2020) with relatively disappointing outcomes. Considering their central role in sarcomere mechanics, Ca^2+^ handling and myofilament Ca^2+^ sensitivity stand out as targets to treat cardiac disease. L-type Ca^2+^ channel blockers are well established to treat intracellular calcium overload in cardiovascular disease (Rosing et al. 1979; Akhtar et al. 1989; Udelson et al. 1989; Gistri et al. 1994; Ho et al. 2015). Promising results to reduce myofilament Ca^2+^ sensitivity have been obtained with catechins, contained in green tea, in an animal model of genetic HCM (Adhikari and Wang 2004). Furthermore, molecules developed to inhibit calmodulin (Hidaka et al. 1980; Silver et al. 1986; Osawa et al. 1998) hold promise to exert a similar inhibitory effect on myofilaments due to the structural homology between calmodulin and cTnC. In the heart, the positive inotropic effect of β-adrenergic signaling is accompanied by reduced myofilament Ca^2+^ sensitivity via PKA phosphorylation of cTnI (Ser23/24) (Zhang et al. 1995; Kentish et al. 2001). β-Adrenergic receptor antagonists (β-blockers) have been used for decades to treat a variety of cardiac diseases, including hypertension, heart failure, cardiac arrhythmias, and myocardial infarction. Among the β-blockers used in clinical therapy, nebivolol has been reported to additionally desensitize cardiac myofilaments (Zeitz et al. 2000). Clinical trials using small molecules to change sarcomere mechanics are promising and include the selective cardiac myosin activator omecamtiv mecarbil to treat systolic heart failure (Teerlink et al. 2021) and the selective myosin inhibitor mavacamtem to treat obstructive HCM (Olivotto et al. 2020). Mavacamtem acts on myosin and stabilizes the SRX configuration thus reducing hypercontractility (Kawas et al. 2017; Anderson et al. 2018). Cardiac myosin isoform ratio is known to change in favor of β-MyHC in disease; thus, strategies to counteract this isoform shift could be of benefit. The RNA transcript of the ancient myosin 7b (MYH7b) — non-translated in mammalian hearts (Rossi et al. 2010; Warkman et al. 2012; Lee et al. 2019; Peter et al. 2019) — includes a long non-coding RNA that has been demonstrated capable of regulating the expression ratio of α-MyHC and β-MyHC in human iPSC-derived cardiomyocytes, and thus may represent a novel therapeutic target (Broadwell et al. 2021).

### Targeting passive forces

Titin is an excellent target to address cardiac diseases related to altered passive tension. High sarcomere passive stiffness due to low PKG titin phosphorylation is found in some patients with heart failure. PKG activity depends on the levels of cyclic guanosine monophosphate and may be increased via inhibition of phosphodiesterases. However, treatments of heart failure patients with the phosphodiesterase-5 inhibitor, sildenafil (Redfield et al. 2013; Hoendermis et al. 2015) did not improve outcomes. Promising results have been obtained by inhibiting phosphodiesterase-9A in diseased human tissues (Lee et al. 2015), although its expression in failing human cardiomyocytes remains uncertain (Li et al. 2019). Other studies have shown that the specific stimulation of β_3_ adrenergic receptors may exert beneficial effects incardiomyocytes by increasing PKG activity (Gauthier et al. 1998; Hammond and Balligand 2012). PKG phosphorylates the same PKA site in cTnI (Ser23/24) reducing myofilament Ca^2+^ sensitivity (Lee et al. 2010). Thus, activation of β_3_ adrenergic receptor would conveniently decrease myofilament Ca^2+^ sensitivity while improving sarcomere relaxation.

In a mouse model of heart failure with preserved ejection fraction, administration of metformin, used in patients to treat type 2 diabetes, improved diastolic function based on the phosphorylation of a PKA site on N2B domain of titin (Slater et al. 2019). An alternative strategy to change passive tension builds on RBM20 to target titin isoform expression and has been explored in different experimental models of diastolic dysfunction (Methawasin et al. 2014; Hinze et al. 2016; Methawasin et al. 2016). Improved ventricular filling and exercise capacity, reduced end-diastolic pressure, and increased expression of hypertrophic genes were associated with negative effects on the Frank–Starling mechanism, force generation, and slower cross-bridge kinetics (Pulcastro et al. 2016), highlighting the tight connection between passive and active forces in the sarcomere. Together with titin, the extracellular matrix represents a major contributor of cardiac passive tension. Fibrosis, the excessive deposition of collagen and altered composition of the extracellular matrix, is a hallmark of several cardiac diseases. Although we focused this review on forces originating from the sarcomere, combating cardiac fibrosis remains an essential clinical intervention (Travers et al. 2016). Notably, fibrosis can induce a switch to the more compliant N2BA titin isoform to partially counteract increased collagen-based stiffness (Neagoe et al. 2002; Makarenko et al. 2004; Nagueh et al. 2004; Hamdani et al. 2010). Although non-sarcomeric in nature, antifibrotic therapy could affect titin isoform composition. Advances in biomaterial sciences to control the extracellular stiffness (Walker et al. 2021; Jian et al. 2014; Crocini et al. 2020; Shimkunas et al. 2021), could help dissecting the crosstalk between the cardiac sarcomere and the extracellular matrix and their relative contribution to cardiac passive tension.

## Concluding remarks

Cardiac diseases remain an enormous burden and unresolved medical problem worldwide. The need for novel therapeutic approaches and interventions is obvious, and targeting cardiac sarcomeres is a powerful and effective strategy to combat cardiac disorders. Significant progress has been made toward understanding cardiac sarcomere structure and function, but a comprehensive and detailed picture is still missing — starting with the complete survey of the dynamic sarcomeric proteome, its interactions and adaptation in health and disease.
