# Correction to: Cardiac sarcomere mechanics in health and disease

**DOI:** 10.1007/s12551-023-01086-1

**Published:** 2023-07-01

**Authors:** Claudia Crocini, Michael Gotthardt

**Affiliations:** 1 Max Delbrück Center for Molecular Medicine in the Helmholtz Association (MDC), Neuromuscular and Cardiovascular Cell Biology, Berlin, Germany; 2 German Center for Cardiovascular Research (DZHK) Partner Site Berlin, Berlin, Germany; 3 BioFrontiers Institute & Department of Molecular and Cellular Development, University of Colorado Boulder, Boulder, USA; 4 Charité-Universitätsmedizin Berlin, 10117 Berlin, Germany

**Correction to: Biophysical Reviews (2021) 13:637–652**

**https://doi.org/10.1007/s12551-021-00840-7**

In Fig. 2B of this article’s original version, some of the mechanisms for changing titin stiffness were accidentally inverted. The corrected version of Fig. 2B is shown below.

**Fig. 2:**
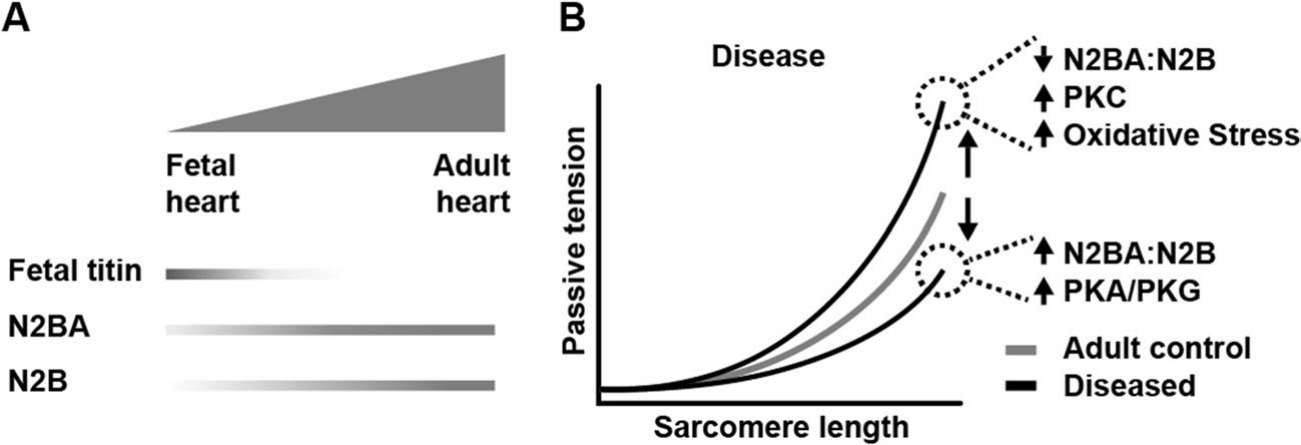
Titin-based passive tension. **A** Developmental changes in titin isoform expression facilitate the transition from fetal to adult force generation. **B** The relationship of sarcomere length and passive tension changes in disease via posttranslational modifications (protein kinases PKA, PKC, PKG acting on the titin spring region) and changes in titin isoform expression (N2BA to N2B titin isoform ratio)

